# Multispectral Pulsed Photobiomodulation Enhances Re-Epithelialization via Keratinocyte Activation in Full-Thickness Skin Wounds

**DOI:** 10.3390/cells14181415

**Published:** 2025-09-10

**Authors:** Joo Hyun Kim, Delgerzul Baatar, Myung Jin Ban, Ji Won Son, Jihye Choi, Chan Hee Gil, Min-Kyu Kim, Sung Sik Hur, Jung Eun Kim, Yongsung Hwang

**Affiliations:** 1 Soonchunhyang Institute of Medi-Bio Science, Soonchunhyang University, Asan 31538, Republic of Korea; noirsky@naver.com (J.H.K.); 20247491@sch.ac.kr (D.B.); pearl3717@naver.com (J.W.S.); cwg0311@naver.com (J.C.); gavyek@gmail.com (M.-K.K.); sstahur@gmail.com (S.S.H.); 2 Department of Otorhinolaryngology-Head and Neck Surgery, Soonchunhyang University, Cheonan Hospital, Cheonan 31151, Republic of Korea; mjbanent@schmc.ac.kr (M.J.B.); heladoo81@gmail.com (C.H.G.); 3 Department of Integrated Biomedical Science, Soonchunhyang University, Asan 31538, Republic of Korea; 4 Department of Dermatology, Soonchunhyang University, Cheonan Hospital, Cheonan 31151, Republic of Korea

**Keywords:** full thickness wound model, low-level light therapy (LLLT), cell migration, re-epithelialization, keratinocytes

## Abstract

Chronic wound healing is a complex and tightly regulated process requiring coordinated epithelial and stromal regeneration. Photobiomodulation (PBM) using low-level red light-emitting diode (LED) therapy has emerged as a non-invasive approach to enhancing skin repair. In this study, we evaluated the therapeutic efficacy of a pulsed, multi-wavelength LED system on full-thickness excisional wound healing in a normal murine model. Daily LED treatment significantly accelerated wound closure, promoted re-epithelialization, and improved dermal architecture. Histological and immunohistochemical analyses revealed enhanced epidermal stratification, reduced inflammation, and improved collagen organization. Molecular profiling demonstrated increased expression of proliferation marker Ki67, keratins CK14 and CK17, and extracellular matrix-related genes including MMPs, Col1a1, and Col3a1. In vitro assays using HaCaT keratinocytes showed accelerated scratch wound closure and cytoskeletal remodeling following PBM exposure. These findings suggest that pulsed PBM promotes coordinated epithelial regeneration and matrix remodeling, highlighting its potential as a tunable and effective therapeutic modality for accelerating cutaneous wound healing under physiological conditions.

## 1. Introduction

Skin wound healing is dynamic and tightly coordinated process that proceeds through sequential yet overlapping phases, including hemostasis, inflammation, cell proliferation, migration, and extracellular matrix (ECM) remodeling [1,2]. In the initial inflammatory stage, neutrophils and circulating monocytes rapidly infiltrate the wound site. Monocytes subsequently differentiate into macrophages, which play central roles in debris clearance and orchestration of the immune response [3]. As repair progresses, keratinocytes, fibroblasts, endothelial cells, and immune cells migrate into the wound bed, guided by spatial and temporal gradients of cytokines and growth factors that regulate tissue repair [4,5,6]. The proliferative phase is marked by cell expansion, angiogenesis, and deposition of new ECM, processes that re-establish both dermal and epidermal structure [2]. Among the signaling molecules involved, transforming growth factor-β1 (TGF-β1) is a pivotal regulator, controlling macrophage activity and ECM turnover during this stage [7].

To promote optimal wound healing, a number of therapeutic interventions have been explored, including dressings, negative pressure therapy, electrical stimulation, hyperbaric oxygen, and nanomaterial-based platforms [8]. However, several of these approaches are limited by invasiveness, high cost, potential cytotoxicity, or poor patient compliance [9,10]. Therefore, the development of non-invasive, cost-effective, and biologically safe alternatives remains a critical clinical need.

In this context, low-level light therapy (LLLT), particularly in the red and near-infrared (NIR) spectrum delivered via laser or LED, has emerged as a promising non-pharmacological intervention for promoting skin regeneration [11,12,13]. LLLT is capable of stimulating key cellular activities, including proliferation, migration, and matrix production, without direct contact or systemic drug administration. In parallel, NIR-responsive nanomaterials have garnered interest in cancer therapy due to their ability to selectively activate intracellular pathways with minimal invasiveness [14]. These insights emphasize the therapeutic potential of NIR wavelengths for deeper tissue penetration and selective modulation of cellular signaling, thereby supporting the mechanistic rationale behind our multispectral PBM approach.

Red light has been reported to enhance keratinocyte and fibroblast proliferation and migration, thereby promoting re-epithelialization and matrix remodeling [15,16,17]. Photobiomodulation (PBM) using LED platforms further amplifies these regenerative effects by modulating mitochondrial activity, activating intracellular signaling cascades, and promoting cytoskeletal organization and anti-inflammatory responses [18,19]. Several studies support wavelength-specific effects of PBM; for instance, LEDs in the 630–830 nm range have been shown to accelerate epithelialization and collagen synthesis in preclinical wound models [20]. Similarly, 660 nm red light can promote cell viability and matrix protein production through activation of mitochondrial chromophores such as cytochrome c oxidase, leading to enhanced ATP (adenosine triphosphate) synthesis and cellular activity [21,22].

Beyond traditional parameters, emerging work highlights the significance of PBM-induced regulation of focal adhesion signaling and immune-ECM coordination during wound repair. For example, engineered hydrogels capable of modulating oxygen or redox environments have been used to promote vascularization and macrophage polarization, improving healing outcomes [23,24]. While these biomaterial-based strategies underscore the importance of the local microenvironment, there remains a need for material-free approaches that can activate endogenous repair pathways in a controlled manner.

In this study, we investigated the therapeutic potential of a multispectral, pulsed LED-based PBM platform in promoting full-thickness skin regeneration under physiological (non-pathological) conditions using a wild-type (WT) mouse model. Building on our previous findings in a diabetic wound model, where the same PBM system accelerated tissue repair by enhancing focal adhesion signaling and extracellular matrix (ECM) remodeling, we sought to examine its efficacy in a non-diseased context [17]. The device combines four distinct wavelengths (670, 780, 830, and 910 nm), selected to synergistically target multiple regenerative pathways through wavelength specific cellular activation. Specifically, we focused on evaluating how PBM modulates keratinocyte migration in vitro using HaCaT cells and promotes re-epithelialization in vivo. Re-epithelialization was assessed by immunohistochemical analysis of proliferation and differentiation markers, including Ki-67, cytokeratin 14 (CK14), and cytokeratin 17 (CK17). By combining in vitro scratch assays, gene and protein analyses, and in vivo wound healing evaluations, we aimed to clarify the mechanisms by which PBM enhances keratinocyte dynamics and epithelial restoration. These findings contribute to a deeper mechanistic understanding of light-driven wound healing and support the application of PBM as a non-invasive tool to enhance skin regeneration.

## 2. Materials and Methods

### 2.1. Multispectral PBM System Setup

The light source used in this study was a multispectral photobiomodulation (PBM) unit (PMD-FA240, Ptech Corp., Pyeongtaek, Republic of Korea). The instrument is equipped with four LEDs that emit at 670 nm (red) and at 780, 830, and 910 nm in the near-infrared range. Their corresponding power outputs were 13.6 mW, 3.71 mW, 61.1 mW, and 11.1 mW, which translate to energy delivery rates of 0.0136, 0.00371, 0.0611, and 0.0111 J/s, respectively. The diodes were programmed to operate in a pulsed sequence consisting of 1400 µs illumination followed by a 200 µs pause. For both cell culture and animal experiments, irradiation was applied for 20 min over an area of 1 cm^2^, while the angle of incidence and distance from the sample were kept constant. Under these conditions, the system provided an average energy density of approximately 94 J/cm^2^ per treatment session. The choice of irradiation settings (pulse cycle, exposure time, and fluence) was based on device specifications and guided by our earlier work with the same platform, where similar conditions were shown to facilitate wound repair through focal adhesion-related mechanisms and extracellular matrix remodeling [17].

### 2.2. Keratinocyte Culture and PBM Exposure Protocol

Human immortalized keratinocytes (HaCaT; cat# T0020001, AddexBio, San Diego, CA, USA) were cultured in Dulbecco’s Modified Eagle Medium (DMEM; #10-013-CV, Corning, NY, USA) containing 10% fetal bovine serum (FBS; #35-079-CV, Corning, NY, USA) and 1% penicillin–streptomycin (P/S; #1514022, Corning, NY, USA). HaCaT cells were cultured at 37 °C in 5% CO_2_. Cells were plated at a density of 3 × 10^5^ per well on standard tissue culture plates. Before PBM exposure, cells were gently washed with phosphate-buffered saline (PBS; #70011069; Thermo Fisher Scientific, Waltham, MA, USA). To avoid dehydration and reduce background absorbance due to phenol red, PBS supplemented with 2% FBS was used during irradiation. Photobiomodulation was performed using a multispectral PBM device capable of emitting pulsed light at 670, 780, 830, and 910 nm. The system was operated with a pulse cycle of 1400 µs illumination followed by a 200 µs pause. Each treatment lasted 20 min, and the PBM source was kept at a fixed vertical distance from the plate surface to ensure consistent irradiation and reproducible energy delivery across the wells.

### 2.3. Assessment of Keratinocyte Proliferation

Cell proliferation was evaluated using a standard MTT colorimetric assay. Following PBM exposure, HaCaT cells were incubated for up to 48 h, and metabolic activity was measured at 24 h intervals. At each time point, the culture medium was replaced with MTT solution (3-(4,5-dimethylthiazol-2-yl)-2,5-diphenyltetrazolium bromide; #M6494; Invitrogen, Waltham, MA, USA), and cells were kept for 2 h in the dark to facilitate formazan crystal production. The resulting crystals were subsequently dissolved in 200 µL in dimethyl sulfoxide (DMSO; #D4540, Sigma-Aldrich, St. Louis, MO, USA), and absorbance was measured spectrophotometrically. Each experimental condition was tested in triplicate.

### 2.4. In Vitro Migration Analysis

Cells were plated into a two-well culture insert (#80206; ibidi, Gräfelfing, Germany) and grown until a confluent uniform monolayer reached confluence. The insert was then removed to generate a defined acellular gap, after which the cells were immediately exposed to LED irradiation for 20 min. Wound closure was monitored at 0, 24, 48, and 72 h using an EVOS imaging system, and gap area was quantified with MATLAB (MathWorks, Natick, MA, USA; version R2023b). The wound closure (%) was determined by the following formula:

Wound closure (%) = [(initial area − remaining area)/initial area] × 100%

### 2.5. RNA Extraction and qPCR Analysis

For both HaCaT cells and mouse wound tissues, total RNA was obtained with TRIzol™ reagent (#15596018; Invitrogen, Waltham, MA, USA). For tissue specimens, homogenization was performed with a bead beater and bead-containing homogenization tubes (#BC-1002(c1); Cat#BCS-BT13722; Scinomics, Inc., Daejeon, Republic of Korea). Concentration and purity of RNA were assessed with a NanoPhotometer N60 (Implen Scientific Inc., Munich, Germany). cDNA was synthesized with ReverTra Ace™ qPCR RT Master Mix supplemented with gDNA removal step (#FSQ-301; TOYOBO, Osaka, Japan) on a T100™ Thermal Cycler (Bio-Rad, Hercules, CA, USA). Quantitative PCR was carried out with SYBR Green master mix (#F0924K; TOYOBO, Osaka, Japan) on a QuantStudio platform (Applied Biosystems, Waltham, MA, USA). Gene expression levels were quantified with the 2^−∆∆Ct^ method, normalizing to GAPDH as the reference gene. Relative expression levels are presented as fold change versus control. The sequences of the primers are listed in Table 1 and Table 2.

### 2.6. Protein Extraction and Western Blotting

Whole-cell lysates were prepared using RIPA buffer #EBA11491, Elpis Biotech, Daejeon, Republic of Korea) supplemented with protease and phosphatase inhibitors (Sigma-Aldrich). Protein concentration was measured with the Pierce BCA Protein Assay Kit (#23225, Thermo Fisher Scientific). Equal amounts of protein were separated by SDS-PAGE and transferred onto polyvinylidene difluoride (PVDF) membranes (Pall Corporation, Port Washington, NY, USA). Membranes were blocked for 1 h at room temperature in 5% skim milk (#SKI400.500, BioShop, Burlington, ON, Canada) prepared in Tris-buffered saline with 0.1% Tween-20 (TBST; #P1379-100ML, Sigma-Aldrich), followed by overnight incubation at 4 °C with primary antibodies diluted according to the manufacturer’s instructions. After washing in TBST, membranes were probed with HRP-conjugated secondary antibodies for 1 h at room temperature. Protein bands were visualized using Amersham ECL Prime detection reagent (#RPN2235, Cytiva, Marlborough, MA, USA) and captured on an Amersham Imager 600 (GE Healthcare, Chicago, IL, USA). Band intensity was quantified using ImageJ software (RRID:SCR_003070). Original, uncropped, and unadjusted raw western blot images are provided in the Supplementary Information (Figure S1).

### 2.7. In Vivo Photobiomodulation Treatment in Murine Wound Healing

Male BALB/c mice, seven weeks of age (*n* = 14), were purchased from Orient Bio Corp. (Seongnam, Republic of Korea) and acclimated for 1 week prior to experimentation. All procedures were performed under approval from the Institutional Animal Care and Use Committee of Soonchunhyang University (approved protocol no. SCH22-0033). Under general anesthesia induced with isoflurane (Ifran; Hana Pharm Co., Ltd., Hwa-Sung, Republic of Korea), the dorsal skin of each mouse was shaved and disinfected. Two bilateral full-thickness excisional wounds (8 mm in diameter) were created simultaneously using sterile biopsy punches (#BP-80F, Kai Medical, Seki City, Japan), with an inter-wound distance of approximately 1.5 cm between wound centers to minimize potential wound-to-wound interference [25]. This simultaneous bilateral approach is widely used in murine wound-healing models and enabled each mouse to serve as its own internal control, thereby minimizing inter-individual variability. Animals were randomly assigned to two endpoint cohorts (*n* = 7 per group at Day 7 and Day 15 post-injury). For each mouse, the wound on the left side received daily LED irradiation (20 min per session), whereas the contralateral wound on the right side was left untreated as the paired control. LED treatment parameters, including the four-wavelength pulsed output and fluence, were consistent with those described in Section 2.1. Mice were sacrificed at their designated time points for histological and molecular analyses.

### 2.8. Quantitative Assessment of Wound Closure

Digital images of the wound sites were captured every other day starting from Day 0 (the day of injury) until Day 15. Wound areas were quantified using MATLAB software to calculate the percentage of closure over time. The extent of healing was determined by comparing the current wound area to its initial size, using the following equation [26]:

Closed area (%) = [(initial wound area − current wound area)/initial wound area] × 100%.

### 2.9. Tissue Processing and Histological Evaluation

Collected skin tissues were fixed in 4% paraformaldehyde (PFA) at 4 °C for 24 h, then dehydrated through graded ethanol, cleared with xylene, and embedded in paraffin. Paraffin blocks were sectioned into 4 μm slices using a microtome and subjected to hematoxylin and eosin (H&E), Masson’s trichrome, or immunohistochemistry (IHC) for histological evaluation. Following the staining procedures, a board-certified histopathologist performed all histological analyses based on the International Harmonization of Nomenclature and Diagnostic Criteria (INHAND) methodology to ensure the consistency and accuracy of the results (Table 3) [27]. For H&E staining, sections were treated with hematoxylin for 10 min, rinsed in running tap water for 3 min, and then counterstained with eosin for 1 min 20 s. After graded ethanol dehydration and xylene clearing, slides were mounted for microscopic evaluation. Masson’s trichrome staining involved deparaffinization, fixation in Bouin’s solution for 60 min, and washing for 10 min under running water. Sections were then sequentially stained with Weigert’s iron hematoxylin (10 min), Biebrich scarlet-acid fuchsin (10 min), phosphomolybdic/phosphotungstic acid (10 min), aniline blue (10 min), and 1% acetic acid (5 min). For IHC analysis, paraffin-embedded sections were deparaffinized, subjected to heat-mediated antigen retrieval, and blocked with serum. Samples were incubated overnight at 4 °C with primary antibodies against cytokeratin 14 (CK14; sc-53253, Santa Cruz Biotechnology, Dallas, TX, USA) and cytokeratin 17 (CK17; ab109725, Abcam, Cambridge, UK). Following washes, HRP-conjugated secondary antibodies were applied, and nuclei were counterstained with Mayer’s hematoxylin. All histological slides were independently reviewed by a board-certified pathologist, and diagnostic reports were provided to ensure objective and reliable interpretation of epidermal and dermal regeneration features.

### 2.10. Stastical Analysis

All results are expressed as mean ± standard error of the mean (SEM). Statistical comparisons were performed using Student’s *t*-test or one-way analysis of variance (ANOVA) with GraphPad Prism software (version 10; GraphPad Software, San Diego, CA, USA). Levels of significance were defined as follows: ns (not significant), *p* > 0.05; * *p* < 0.05; ** *p* < 0.01; *** *p* < 0.001.

## 3. Results

### 3.1. PBM Enhances Keratinocyte Migration While Maintaining Proliferative Capacity

**Figure 1:**
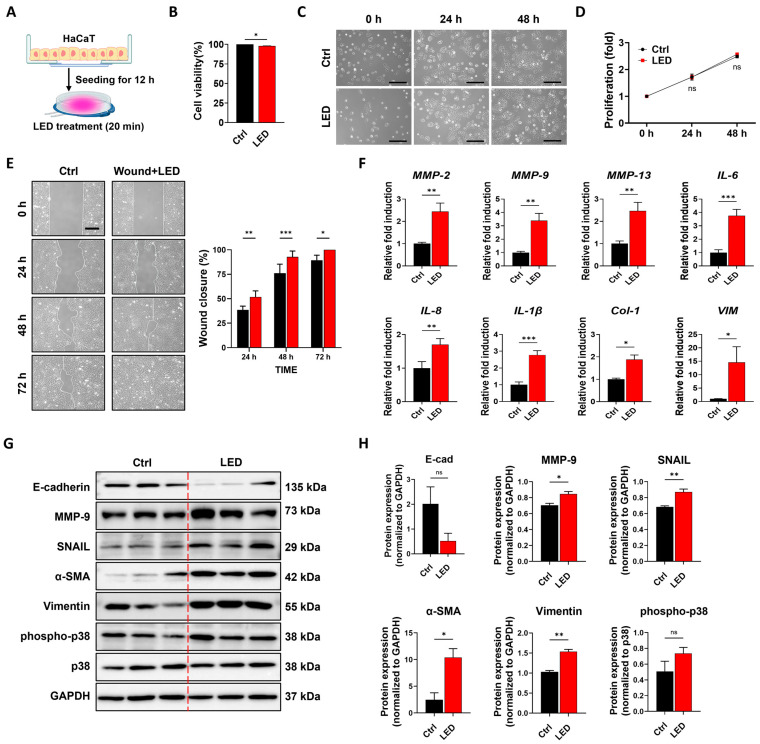
Multispectral pulsed PBM stimulation promotes keratinocyte migration without impairing cell viability. (**A**) Schematic illustration of the PBM treatment procedure for HaCaT cells cultured in a standard cell culture dish. (**B**) Cell viability assay showing that 24 h of multispectral pulsed PBM exposure does not induce phototoxicity. (**C**) Representative phase-contrast images showing time-dependent morphological changes in HaCaT cells over 48 h with or without PBM treatment. Scale bar = 100 µm. (**D**) Quantification of cell proliferation using the MTT assay at 24 h intervals for 48 h post-PBM exposure. (**E**) Representative images of scratch wound healing in HaCaT cells with or without PBM treatment. Scale bar = 300 µm. Wound closure (%) was quantified using MATLAB by calculating the remaining wound area relative to the initial gap width. Statistical significance: ns (not significant), * *p* < 0.05, ** *p* < 0.01, *** *p* < 0.001 (Two-way ANOVA). (**F**) Quantitative real-time PCR analysis of gene expression in HaCaT cells 12 h after LED irradiation. The expression levels of genes related to ECM remodeling (MMP2, MMP9, MMP13), inflammatory response (IL6, IL8, IL1β), ECM synthesis (Col-1), and cell migration (Vimentin) were measured. GAPDH was used as an internal control. Data are presented as the mean ± SEM (*n* = 3). (**G**) Immunoblot analysis of HaCaT cells following PBM exposure, evaluating the expression levels of E-cadherin, MMP-9, Snail, α-SMA, vimentin, p38, and phospho-p38. GAPDH served as a loading control. (**H**) Densitometric quantification of immunoblot bands. Data are shown as mean ± SEM. * *p* < 0.05, ** *p* < 0.01, *** *p* < 0.001.

To examine the biocompatibility of PBM, cells were cultured and exposed to multispectral pulsed irradiation (Figure 1A). A cell viability assay conducted 24 h post-treatment confirmed that the multispectral pulsed LED protocol did not induce cytotoxicity (Figure 1B). To further assess whether repeated stimulation influences cellular metabolism and growth, HaCaT cells received daily PBM treatment for 48 h, and metabolic activity was evaluated using the MTT assay. Throughout the 48 h period, PBM-treated HaCaT cells exhibited metabolic activity comparable to untreated controls (Figure 1C,D), suggesting that PBM did not impair proliferative capacity or cause growth arrest under the tested conditions.

Given that keratinocyte migration plays a critical role in wound re-epithelialization, we next investigated the impact of PBM on this process. A scratch wound assay revealed that PBM treatment accelerated the wound closure rate in HaCaT monolayers. At 48 h and 72 h post-scratch, PBM-treated cultures displayed significantly enhanced wound closure compared to controls (Figure 1E). Quantitative image analysis using MATLAB confirmed this effect, indicating that multispectral pulsed light stimulation promotes collective keratinocyte migration in vitro.

To explore potential molecular mechanisms underlying enhanced migration, we evaluated the mRNA expression of genes associated with inflammation, ECM remodeling, and motility 12 h after PBM exposure. In HaCaT keratinocytes, PBM stimulation significantly increased the expression of *IL-6*, *IL-8*, *IL-1β*, *MMP-2*, *MMP-9*, and *MMP-13*, genes associated with pro-inflammatory signaling and ECM degradation, as well as *Col1a1*, a key ECM synthesis marker. Similarly, in fibroblasts, PBM exposure upregulated *IL-6*, *IL-8*, *MMP-9*, and *MMP-13*, along with *Col1a1* and the motility-associated gene *Vimentin* (Figure 1F).

To validate these transcriptional changes at the protein level, Western blot analysis was performed on HaCaT lysates collected post-PBM treatment. As shown in Figure 1G,H, PBM stimulation led to decreased E-cadherin expression (~0.4-fold), suggesting loss of cell–cell junctions often associated with increased motility. Concurrently, α-smooth muscle actin (α-SMA) and vimentin were upregulated approximately 5-fold and 1.5-fold, respectively, supporting cytoskeletal remodeling and mesenchymal activation. MMP-9 and Snail expression revealed statistically significant increases, indicating that PBM may facilitate epithelial remodeling and migratory activity through partial activation of pathways linked to epithelial–mesenchymal transition (EMT). However, given the complexity of wound-healing networks, these results should be interpreted with caution, and future studies incorporating additional EMT markers will be necessary for definitive validation. Similarly, total and phosphorylated p38 (pp38) were moderately increased (~1.1- to 1.2-fold), without reaching statistical significance.

Collectively, these findings suggest that PBM enhances the migratory capacity of keratinocytes without compromising cell viability or proliferation. This migratory enhancement is accompanied by transcriptional and protein-level modulation of wound-healing–related markers involved in inflammation, ECM remodeling, and cytoskeletal dynamics.

### 3.2. PBM Accelerates Early Wound Closure and Promotes Re-Epithelialization In Vivo

**Figure 2:**
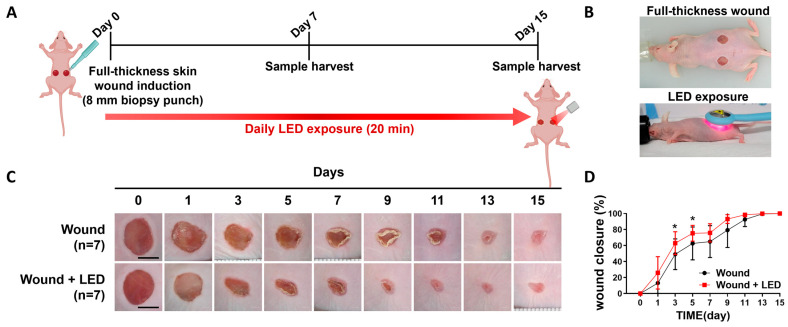
Multispectral pulsed PBM accelerates wound closure in a full-thickness skin defect model. (**A**) Schematic representation of the in vivo experimental design. Full-thickness dorsal skin wounds (8 mm in diameter) were created on Day 0 using a biopsy punch. Seven-week-old nude mice were exposed to daily PBM (20 min/day), and tissue samples were harvested on Day 7 or Day 15 for analysis. (**B**) Representative images showing the full-thickness wounds and the PBM irradiation procedure in 7-week-old nude mice. (**C**) Representative macroscopic images of wound healing progression at the indicated time points. Scale bars = 5 mm. (**D**) Quantitative analysis of wound closure over time. Wound area was calculated relative to the initial wound size, and closure (%) was plotted for each time point. PBM-treated wounds exhibited significantly greater closure at Days 5–11 compared to untreated controls (* *p* < 0.05). Data are presented as mean ± SEM (*n* = 7 per group).

To investigate the in vivo effects of multispectral pulsed PBM on skin regeneration, we utilized a full-thickness excisional wound model in wild-type mice (Figure 2A). Circular dorsal wounds (8 mm diameter) were created on Day 0 using a biopsy punch, and mice received daily PBM irradiation (20 min/session) over a 15-day period. Wound tissues were collected at Days 7 and 15 for macroscopic and histological evaluation (Figure 2A,B). Compared to untreated controls, the PBM-treated group exhibited markedly improved wound closure. Gross observation revealed faster wound resolution in PBM-exposed mice (Figure 2C), which was further supported by quantitative measurement of wound area; representative images are shown in Figure 2C, while the complete set of macroscopic images is provided in the Supplementary Information (Figure S2, *n* = 7). From Day 5 to Day 11, PBM-treated wounds consistently showed enhanced closure, with a significant increase of 10.96% on Day 7 (75.84% in PBM vs. 64.88% in control, * *p* < 0.05) (Figure 2D). By Day 15, both groups approached near-complete wound closure, indicating that the observed effect of PBM was most prominent during the early-to-mid healing phases. In parallel, histological assessments were conducted on formalin-fixed wound tissues collected at Days 7 and 15 to assess parameters relevant to skin repair, including scab formation, degree of re-epithelialization, dermal collagen remodeling, neovascularization, and inflammatory cell infiltration. These analyses were designed to evaluate the structural and cellular responses contributing to improved healing following PBM treatment.

### 3.3. PBM Enhances Epidermal Regeneration and Collagen Remodeling During Wound Healing

**Figure 3:**
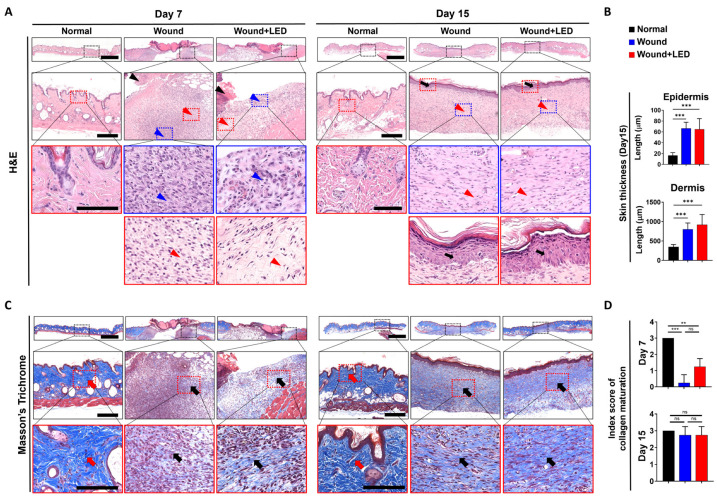
PBM improves wound repair and extracellular matrix remodeling in vivo. Skin wound samples were collected on days 7 and 15 after injury from normal skin, untreated wounds, and PBM-treated wounds. (**A**) Representative hematoxylin and eosin (H&E) images illustrate structural changes in the epidermis and dermis. Insets highlight key histological features, including epidermal necrosis with eschar (black arrowhead), inflammatory cell infiltration (blue arrowhead), dermal fibroblasts (red arrowhead), and newly regenerated epidermis (black arrow). (**B**) Quantification of skin thickness on Day 15, measured from H&E-stained images. (**C**) Representative Masson’s Trichrome–stained sections illustrate collagen distribution, with red arrows indicating native collagen fibers and black arrows marking areas of newly deposited collagen. (**D**) Collagen maturation index score determined from Masson’s Trichrome staining on Days 7 and 15. Scale bars are indicated for each panel: 500 µm (overview), 100 µm (first insets), and 100 µm (second insets, red box). Data are presented as mean ± SEM. Statistical significance: ns, *p* > 0.05; ** *p* < 0.01; *** *p* < 0.001.

To evaluate the effects of multispectral PBM on tissue regeneration, full-thickness skin samples were harvested on Days 7 and 15 and analyzed via hematoxylin and eosin (H&E) and Masson’s Trichrome staining (Figure 3A,C). H&E staining of untreated wounds revealed incomplete re-epithelialization, disorganized dermal structure, and dense inflammatory infiltration, indicating delayed healing. In contrast, PBM-treated wounds exhibited more continuous and stratified epidermal layers, reduced inflammatory cell presence, and improved dermal organization by Day 15 (Figure 3B). Masson’s Trichrome staining further demonstrated greater collagen deposition and more organized fiber architecture in PBM-treated wounds compared to controls (Figure 3C). Quantitative analysis confirmed a significant increase in both epidermal and dermal thickness in the PBM group by Day 15, suggesting enhanced epithelial restoration and dermal matrix formation. A semi-quantitative collagen maturation score revealed significantly improved collagen organization in the PBM group at Day 7, indicating earlier initiation of matrix remodeling. Histopathological assessment also showed that by Day 7, PBM-treated wounds exhibited reduced necrotic crusts, improved epithelial continuity, and enhanced fibroblast-like cell infiltration relative to untreated wounds. By Day 15, PBM-treated wounds displayed near-complete epithelial coverage, more densely packed and aligned dermal collagen fibers, and decreased inflammatory infiltration (Figure 3D). Overall, these results indicate that PBM supports re-epithelialization and promotes dermal remodeling during wound healing by facilitating early tissue regeneration, modulating inflammation, and enhancing collagen matrix maturation.

**Table 3:** The histopathological findings at both day 7 and day 15.

Skin	Groups		Day 7			Day 15		
			Normal	Wound	Wound + LED	Normal	Wound	Wound + LED
	No. of Animals		2	4	4	2	4	4
Epidermis	Necrosis with crust	++	0	0	2	0	0	0
		+++	0	3	2	0	0	0
	Regeneration	+	0	3	3	0	0	0
		++	0	0	1	0	3	2
		+++	0	0	0	0	1	2
Dermis	Maturation, fibroblast to fibrocyte	±	0	3	0	0	0	0
		+	0	1	2	0	0	0
		++	0	0	2	0	1	2
		+++	0	0	0	0	3	3
	Collagen maturation	±	0	3	0	0	0	0
		+	0	1	3	0	0	0
		++	0	0	1	0	1	1
		+++	2	0	0	2	3	3
	Vascularization	±	0	0	0	0	1	0
		+	0	1	0	0	0	0
	Infiltrate, inflammatory cell	±	0	1	1	0	2	3
		+	0	2	0	0	0	0

Grade: (±) Minimal, (+) Mild, (++) Moderate, (+++) Marked.

### 3.4. PBM Enhances Epidermal Proliferation and Re-Epithelialization in a Murine Full-Thickness Wound Model

**Figure 4:**
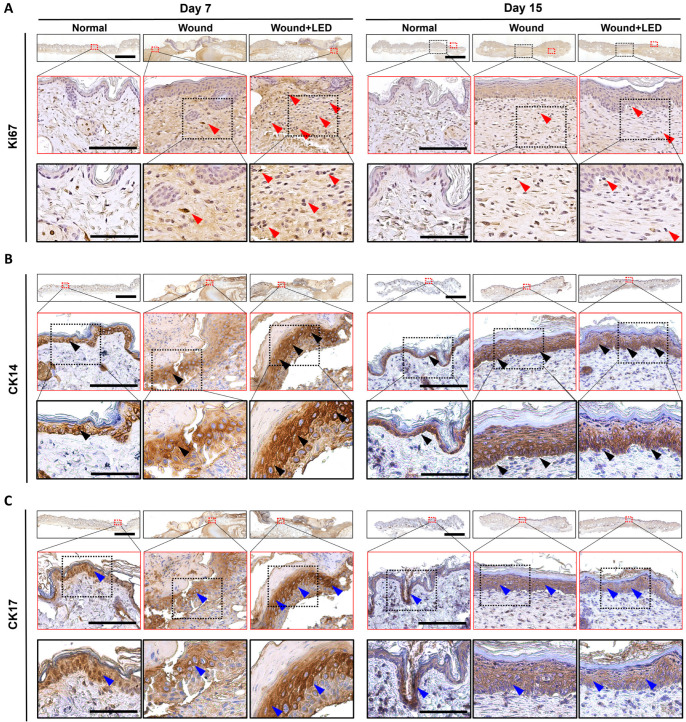
Effects of PBM on epithelial regeneration in a murine full-thickness skin defect model. Histological analysis was performed on wound tissues collected on Days 7 and 15 post-injuries from normal skin (control), untreated wounds, and PBM-treated wounds. (**A**) Ki-67 immunohistochemistry, which identifies proliferating cells in active phases of the cell cycle, was used to examine cellular proliferation (red arrowhead: Ki-67–positive nuclei). (**B**) Cytokeratin 14 (CK14) staining, a marker of basal keratinocytes, was employed to assess re-epithelialization and basal layer organization (black arrowhead: CK14–positive basal epidermal cells). (**C**) Cytokeratin 17 (CK17) staining, indicative of keratinocyte activation during wound remodeling, was analyzed to determine epithelial activation status (blue arrowhead: CK17–positive proliferative keratinocytes). Scale bars: 1 mm (overview), 150 µm (first magnified insets, red boxes), and 300 µm (second magnified insets, black boxes).

Immunohistochemical analyses were conducted to evaluate the effects of PBM on wound healing by examining markers of proliferation and keratinocyte activation in skin tissues harvested at Days 7 and 15 post-injuries. Ki-67 staining, a marker for cellular proliferation, revealed increased numbers of proliferating epidermal cells in PBM-treated wounds compared to untreated wounds at both time points (Figure 4A). At Day 7, Ki-67-positive cells were mainly located in the basal and suprabasal layers of the epidermis in the PBM group, indicating enhanced early proliferative activity. Although proliferation decreased by Day 15, the PBM-treated wounds maintained higher Ki-67 expression relative to untreated wounds, suggesting sustained proliferative capacity during later stages of healing. Immunostaining for cytokeratin 14 (CK14), a basal keratinocyte marker essential for re-epithelialization, demonstrated a stronger and more continuous basal layer in PBM-treated wounds compared to untreated wounds, which showed patchy and reduced CK14 expression (Figure 4B). This difference was particularly notable at Day 7, suggesting that PBM promotes early activation and regeneration of basal keratinocytes. By Day 15, CK14 expression was decreased in the PBM-treated group relative to the wound-only group. This reduction may reflect the completion of epithelial regeneration in the PBM group, where the need for basal keratinocyte activation had subsided. In contrast, the wound-only group maintained higher CK14 expression, likely due to ongoing re-epithelialization and delayed tissue remodeling. Cytokeratin 17 (CK17), a marker of activated keratinocytes induced under acute stress and involved in epithelial cell migration and remodeling, showed robust and widespread expression in PBM-treated wounds at Day 7, extending beyond the basal layer into suprabasal layers (Figure 4C). This reflects an early and active wound repair response facilitated by PBM. By Day 15, CK17 expression was markedly reduced in the PBM-treated group, suggesting that epithelial repair had progressed toward completion and keratinocyte stress response was resolved. In contrast, the wound-only group maintained relatively high CK17 expression at this time point, indicating a delayed or prolonged repair response and ongoing keratinocyte activation. Together, these results suggest that PBM promotes earlier keratinocyte activation, proliferation, and re-epithelialization, thereby accelerating the wound healing process compared to untreated wounds.

### 3.5. PBM Regulates Key Gene Expression Pathways Involved in Wound Healing

**Figure 5:**
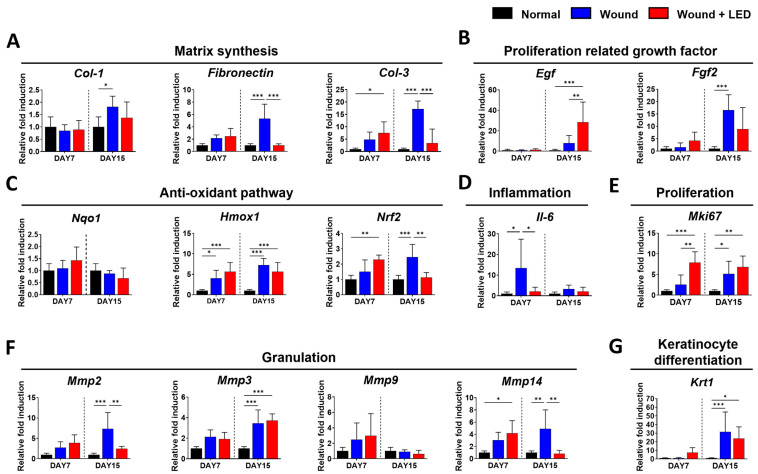
Quantitative real-time PCR analysis of gene expression in mouse skin wound tissues at Days 7 and 15 post-injuries following PBM treatment. Relative mRNA expression levels of genes involved in extracellular matrix (ECM) synthesis (Col1a1, fibronectin, Col3a1) (**A**), growth factors (Egf, Fgf2) (**B**), antioxidant response (Nqo1, Hmox1, Nrf2) (**C**), inflammation (Il6) (**D**), proliferation marker (Mki67) (**E**), granulation-related matrix metalloproteinases (Mmp2, Mmp3, Mmp9, Mmp14) (**F**), and keratinocyte differentiation (Krt1) (**G**) were measured by qPCR. Data are presented as mean ± SEM (*n* = 3). Statistical significance is indicated as * *p* < 0.05, ** *p* < 0.01, *** *p* < 0.001. These results demonstrate that PBM treatment modulates the expression of key genes associated with wound healing in a full-thickness skin defect mouse model. Effects of PBM on wound tissue regeneration in a murine excisional wound model.

To investigate the molecular basis of the regenerative effects of PBM on wound healing, quantitative PCR was performed on skin tissues collected from a full-thickness skin defect model mice at Days 7 and 15 post-injury (Figure 5). Multiwavelength pulsed PBM accelerated wound closure by modulating extracellular matrix (ECM) remodeling, stimulating keratinocyte proliferation and activation, and facilitating reepithelialization. Extracellular matrix (ECM) components, including Col1a1, fibronectin, and Col3a1, were significantly upregulated in untreated wounds at Day 15, reflecting active matrix deposition during wound repair. In contrast, PBM-treated wounds exhibited moderated expression of these genes (Figure 5A,F), suggesting that photobiomodulation promotes a balanced ECM remodeling, preventing excessive fibrosis while supporting proper tissue regeneration. Growth factors such as Egf and Fgf2, which are critical for keratinocyte proliferation and migration during re-epithelialization, were elevated in the PBM-treated group at Day 15 (Figure 5B). This upregulation likely facilitates accelerated re-epithelialization by enhancing cellular activities necessary for epidermal restoration. Oxidative stress markers, including Hmox1 and Nrf2, were also increased in wounds, indicating a stress response. However, PBM treatment further modulated their expression (Figure 5C), suggesting an antioxidative effect that may contribute to a more favorable healing environment by reducing oxidative damage. Pro-inflammatory cytokine Il-6 showed elevated expression in wounds at Day 7, which was significantly attenuated by PBM treatment (Figure 5D). This reduction in inflammatory signaling aligns with the known anti-inflammatory properties of photobiomodulation, which can mitigate chronic inflammation detrimental to healing. Importantly, the proliferation marker Mki67 was significantly increased in PBM-treated wounds at both time points (Figure 5E), indicating enhanced cellular proliferation, a critical step for re-epithelialization and wound closure. Correspondingly, Krt1, a marker of keratinocyte differentiation, was upregulated at Day 15 following PBM treatment (Figure 5G), supporting the notion that photobiomodulation not only stimulates cell proliferation but also promotes differentiation necessary for functional epidermal layer restoration. Finally, matrix metalloproteinases Mmp2, Mmp3, and Mmp14 were elevated during the late stage of healing in untreated wounds, reflecting active ECM remodeling. PBM treatment moderated the expression of Mmp2 and Mmp14 (Figure 5F), suggesting a regulatory role in preventing excessive ECM degradation and favoring controlled matrix remodeling conducive to optimal wound repair. Collectively, these molecular changes demonstrate that PBM facilitates wound healing by promoting balanced ECM remodeling, enhancing keratinocyte proliferation and differentiation, reducing inflammation, and modulating oxidative stress, all of which contribute to accelerated and effective re-epithelialization in full-thickness wounds.

## 4. Discussion

Effective skin regeneration relies on coordinated interactions between keratinocytes and dermal fibroblasts. Keratinocytes initiate re-epithelialization by migrating across the wound surface and proliferating to restore epidermal continuity [28,29]. Simultaneously, fibroblasts contribute to granulation tissue formation by synthesizing extracellular matrix (ECM) proteins such as collagen and fibronectin, which provide mechanical integrity and support for tissue remodeling [30,31]. Keratinocytes also secrete cytokines and growth factors that modulate immune responses and stromal activity, thereby accelerating wound closure [28,32,33,34]. Fibroblasts respond to these cues by migrating into the wound bed and depositing ECM components to support epithelial and vascular regeneration [32,35,36]. As healing progresses, fibroblast-mediated matrix remodeling facilitates granulation tissue maturation and establishes a scaffold for sustained tissue repair.

In vitro assays were conducted using the HaCaT keratinocyte line, a widely adopted model due to its reproducibility and ease of culture. While these cells do not fully capture the characteristics of primary human keratinocytes [37,38], they provide a practical platform for exploring mechanisms of photobiomodulation therapy (PBMT). Our findings offer mechanistic insights into how PBM enhances keratinocyte migration and regenerative activity. Nevertheless, validation using primary keratinocytes and additional in vivo systems will be essential to translate these findings more directly to clinical settings.

In our study, PBM significantly enhanced the migratory capacity of HaCaT keratinocytes in vitro, as evidenced by accelerated scratch wound closure. This finding is consistent with previous work by Sutterby et al. [39], who reported that very low-intensity visible light exposure stimulated HaCaT proliferation and migration without elevating oxidative stress. Notably, red light (660 nm) enhanced mitochondrial activity, suggesting energy-dependent promotion of regenerative processes [39]. During the proliferative phase of wound healing, keratinocytes at the wound margin rapidly migrate and proliferate to restore epithelial continuity, while fibroblasts migrate into the wound bed, proliferate, and differentiate into myofibroblasts, contributing to ECM production and wound contraction [40,41]. The enhanced in vitro keratinocyte migration and in vivo epithelial coverage observed in our study indicate that PBM effectively supports these early regenerative events. It is important to note that murine wound healing can be confounded by panniculus carnosus–mediated contraction; however, our conclusions were based primarily on histological and molecular endpoints (Ki-67, CK14, CK17, ECM remodeling) that specifically reflect re-epithelialization and regeneration, thereby minimizing the risk of misinterpretation due to muscle-driven closure.

Our in vivo results further support a pro-regenerative effect of PBM. At the transcriptional level, genes involved in cell proliferation (Mki67), ECM remodeling (Mmp2, Mmp3, Mmp9), oxidative stress response (Hmox1, Nqo1, Nrf2), and keratinocyte differentiation (Krt1) were upregulated at Day 7 post-injury. Transient induction of MMPs during the remodeling phase is essential for matrix turnover, keratinocyte migration, and resolution of the provisional matrix [42,43], while increased Ki67 expression reflects sustained basal proliferation during re-epithelialization [44,45]. ECM-related genes such as Col1a1 and Col3a1 were also elevated, indicative of active matrix production and remodeling [46]. In parallel, increased expression of vimentin and N-cadherin reflects mesenchymal activation, supporting cytoskeletal plasticity and fibroblast migration [47,48].

Our findings suggest that PBM enhances not only cell motility but also matrix remodeling at both molecular and tissue levels. Although unregulated or excessive MMP expression may contribute to tissue degradation, transient upregulation during the remodeling phase is critical for successful matrix turnover and wound resolution [49,50]. Consistent with this, we observed increased mRNA expression of MMP9, Krt1, and Ki67 at Day 7 post-treatment, suggesting that PBM therapy activates a pro-regenerative transcriptional program. Elevated MMP expressions in this context likely support keratinocyte migration and matrix reorganization, while increased Ki67 reflects enhanced proliferative activity within the wound bed. These molecular changes corresponded with improved histological architecture and reduced fibrotic features at later time points, supporting the notion that PBM facilitates a well-regulated healing response.

Our observations align with previous report demonstrating that a red-laser-based wound therapy device accelerated keratinocyte and fibroblast migration, enhanced collagen and desmoglein expression, and promoted early expression of growth and heat shock factors [16]. Under both physiological and chronic inflammatory conditions, photobiomodulation improved the migratory and synthetic behaviors of skin cells in vitro.

Directed cell migration relies on tightly coordinated cytoskeletal remodeling and focal adhesion dynamics. Actin polymerization at the leading edge and formation of lamellipodia and filopodia drive cellular protrusion, while focal adhesion complexes—including integrins, focal adhesion kinase (FAK), and paxillin—anchor cells to the ECM and convert mechanical cues into intracellular signaling [51,52]. In our previous study, LED treatment promoted FAK phosphorylation and actin remodeling, indicating enhanced focal adhesion turnover and cytoskeletal reorganization in keratinocytes. These observations align with the concept that PBM supports cell motility by strengthening the mechanical and signaling machinery necessary for effective migration [53,54,55].

Histological evaluation further confirmed accelerated healing in PBM-treated wounds. Compared to untreated controls, PBM-treated tissues displayed enhanced epithelial coverage, improved collagen organization, and reduced inflammatory cell infiltration by Day 14. Specifically, a significant decrease in F4/80-positive macrophages at the remodeling stage indicates a timely resolution of the inflammatory response. Although we did not assess macrophage polarization directly, the overall decline in macrophage density and improved tissue morphology suggests a shift toward a resolved, rather than sustained, inflammatory state [43,56,57]. Future studies incorporating immunophenotyping of macrophage subtypes (e.g., CD86, CD206, iNOS, Arg1) will help determine whether PBM also promotes anti-inflammatory M2-like activity.

Statha et al. [58] demonstrated that low-power red laser light (661 nm) promoted wound healing in SKH-hr2 mice through modulation of inflammation and collagen organization. Their findings highlight how energy density and power duration can significantly affect collagen remodeling, echoing our observations of well-organized collagen fibers and reduced inflammatory cell infiltration in PBM-treated wounds.

We also observed a marked reduction in α-SMA expression in the absence of significant changes in COL1 or TGF-β1 expression, suggesting that PBM may suppress myofibroblast differentiation while preserving matrix synthesis [59,60]. These results support prior findings that PBM modulates fibrotic remodeling by targeting cytoskeletal and contractile phenotypes without impairing essential ECM production [13,60]. Importantly, these antifibrotic effects appear to be phase-specific, emerging during the tissue remodeling period rather than early inflammatory or proliferative stages. This timing is consistent with physiological wound resolution and may help prevent excessive scar formation.

In parallel, recent work by Migliario et al. demonstrated that oxidative-stress-dependent mechanisms underlie laser-induced expression of β-defensins in keratinocytes, contributing to their pro-healing and immunomodulatory activity [61]. These findings further support our observation of increased Nrf2, Hmox1, and Nqo1 gene expression following PBM, suggesting that oxidative stress responses may be part of the beneficial downstream signaling cascade.

We also observed marked modulation of key keratinocyte markers during re-epithelialization. CK14 and CK17 were upregulated at Day 7, reflecting basal and activated keratinocyte activity, respectively. Previously, Sperandio et al. showed that LLLT enhanced keratinocyte proliferation and promoted early expression of epithelial differentiation markers including CK14 [29]. Similarly, Patel et al. described the dynamic expression of CK17 and Ki67 in basal and suprabasal layers during acute wound healing, correlating with keratinocyte activation and migration from the wound edge [62]. Taken together, these findings are in line with our immunohistochemical data and transcriptomic results showing early upregulation of CK17 and Ki67 in PBM-treated wounds, suggesting accelerated epithelial repair.

## 5. Conclusions

This study demonstrates that multispectral pulsed PBM offers a material-free and effective strategy to promote cutaneous regeneration. In a full-thickness wound model, PBM markedly accelerated reepithelialization by stimulating keratinocyte activity, as reflected by elevated expression of Ki-67, CK14, and CK17. The combination of multiple wavelengths acted synergistically to enhance epidermal proliferation, temper inflammatory responses, and support organized dermal remodeling, thereby providing new mechanistic insights into how coordinated photobiological cues can drive wound repair. However, some limitations warrant consideration. While our transcriptomic data showed significant gene modulation, further protein-level validation is needed. Additionally, our in vitro models may not fully replicate the complexity of in vivo interactions. A comprehensive analysis of wavelength-specific effects was beyond the scope of this study. Another limitation is that we did not perform microbiological analyses of the wound sites; thus, the potential influence of bacterial flora on healing outcomes remains unassessed. Future research should focus on optimizing treatment parameters, assessing the independent effects of each wavelength, exploring potential microbiological interactions, and further evaluating the translational potential of this technology for clinical wound management.

## Figures and Tables

**Table 1:** The human primer sequences used in this study.

Primer	Sequences (5′–3′)
*GAPDH*_Forward	CACTCCACCTTTGACGC
*GAPDH*_Reverse	GGTCCAGGGGTCTTACTCC
*MMP2*_Forward	GATACCCCTTTGACGGTAAGGA
*MMP2*_Reverse	CCTTCTCCCAAGGTCCATAGC
*MMP9*_Forward	GGGACGCAGACATCGTCATC
*MMP9*_Reverse	TCGTCATCGTCGAAATGGGC
*MMP13*_Forward	TCGTCATCGTCGAAATGGGC
*MMP13*_Reverse	TCGTCATCGTCGAAATGGGC
*IL-6*_Forward	ACTCACCTCTTCAGAACGAATTG
*IL-6*_Reverse	CCATCTTTGGAAGGTTCAGGTTG
*IL-8*_Forward	ACTGAGAGTGATTGAGAGTGGAC
*IL-8*_Reverse	AACCCTCTGCACCCAGTTTTC
*IL-1β*_Forward	ATGATGGCTTATTACAGTGGCAA
*IL-1β*_Reverse	GTCGGAGATTCGTAGCTGGA
*COL-1*_Forward	CAAGACAG TGATTGAATACAAAACCA
*COL-1*_Reverse	ACGTCGAAGCCGAATTCCT
*Vimentin*_Forward	AATCCAAGTTTGCTGACCTCTCTGA
*Vimentin*_Reverse	ACTGCACCTGTCTCCGGTACTC

**Table 2:** The mouse primer sequences used in this study.

Primer	Sequences (5′–3′)
*Gapdh*_Forward	AAGGTCATCCCAGAGCTGAA
*Gapdh*_Reverse	CTGCTTCACCACCTTCTTGA
*Col-1*_Forward	GCT CCT CTT AGG GGC CAC T
*Col-1*_Reverse	CCT TTGTCA GAA TAC TGA GCA GC
*Fibronectin*_Forward	ATGTGGACCCCTCCTGATAGT
*Fibronectin*_Reverse	GCCCAGTGATTTCAGCAAAGG
*Egf*_Forward	AGCATCTCTCGGATTGACCCA
*Egf*_Reverse	CCTGTCCCGTTAAGGAAAACTCT
*Fgf2*_Forward	GCGACCCACACGTCAAACTA
*Fgf2*_Reverse	CCGTCCATCTTCCTTCATAGC
*Nqo1*_Forward	AGGATGGGAGGTACTCGAATC
*Nqo1*_Reverse	AGGCGTCCTTCCTTATATGCTA
*Hmox1*_Forward	AAGCCGAGAATGCTGAGTTCA
*Hmox1*_Reverse	GCCGTGTAGATATGGTACAAGGA
*Nrf2*_Forward	CTGAACTCCTGGACGGGACTA
*Nrf2*_Reverse	CGGTGGGTCTCCGTAAATGG
*Il-6*_Forward	TAGTCCTTCCTACCCCAATTTCC
*Il-6*_Reverse	TTGGTCCTTAGCCACTCCTTC
*Mki-67*_Forward	CTGCCTCAGATGGCTCAAAGA
*Mki-67*_Reverse	GAAGACTTCGGTTCCCTGTAAC
*Mmp2*_Forward	CAAGTTCCCCGGCGATGTC
*Mmp2*_Reverse	TTCTGGTCAAGGTCACCTGTC
*Mmp3*_Forward	GATGAGCACACAACCACACAC
*Mmp3*_Reverse	GGTACAGAGCTGTGGGAAGTC
*Mmp9*_Forward	GGGACGCAGACATCGTCATC
*Mmp9*_Reverse	CCCACATTTGACGTCCAGAGAAGAA
*Mmp14*_Forward	CAGTATGGCTACCTACCTCCAG
*Mmp14*_Reverse	GCCTTGCCTGTCACTTGTAAA
*Krt1*_Forward	TGGGAGATTTTCAGGAGGAGG
*Krt1*_Reverse	GCCACACTCTTGGAGATGCTC

## Data Availability

The raw data supporting the conclusions of this article will be made available by the authors on request.

## Supplementary Materials

The following supporting information can be downloaded at: https://www.mdpi.com/article/10.3390/cells14181415/s1, Figure S1: Original, uncropped, and unadjusted raw images of Western blot used in this study; Figure S2: Macroscopic images of wound healing progression at indicated time points. Scale bars = 5 mm.

## Abbreviations

LED	light-emitting diode
ECM	extracellular matrix
LLLT	Low-level light therapy
TGF-β1	transforming growth factor-beta 1
PBM	photobiomodulation
ATP	adenosine triphosphate
MTT	3-(4,5-dimethylthiazol-2-yl)-2,5-diphenyltetrazolium bromide
α-SMA	alpha smooth muscle actin
