# Supplementary material for: Multispectral Pulsed Photobiomodulation Enhances Re-Epithelialization via Keratinocyte Activation in Full-Thickness Skin Wounds
Source: Cells. 2025 Sep 10;14(18):1415. doi: 10.3390/cells14181415 (PMC12468576; doi:10.3390/cells14181415)
Supplement: Supplementary file 1 [file cells-14-01415-s001.zip › cells-3834051-supplementary.pdf]

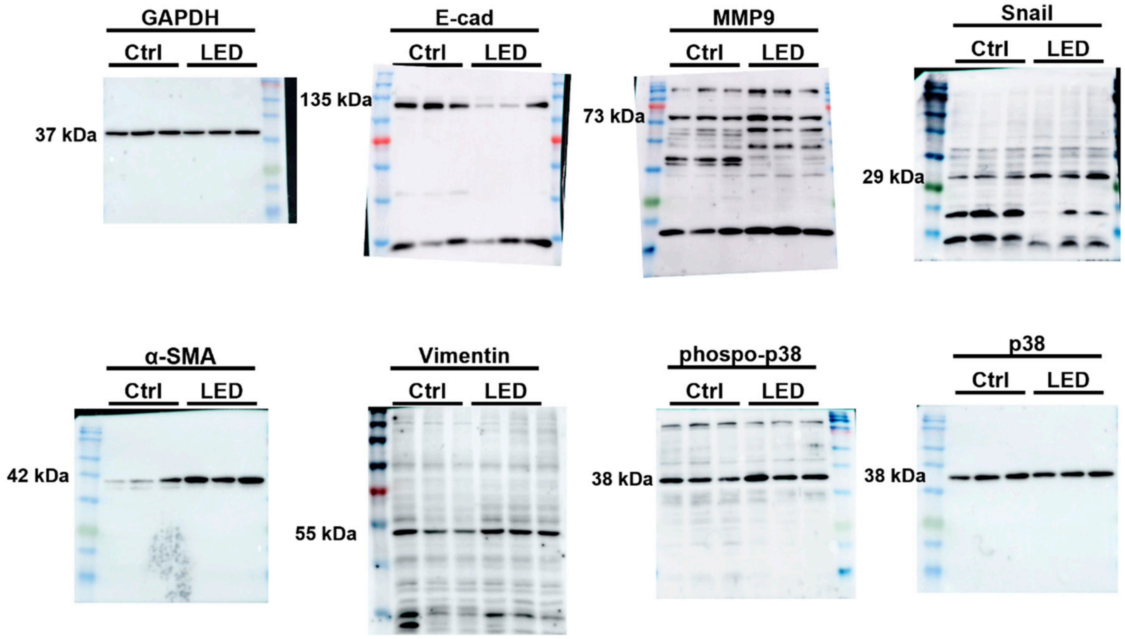

Figure S1. Original, uncropped, and unadjusted raw images of Western blot used in this study.

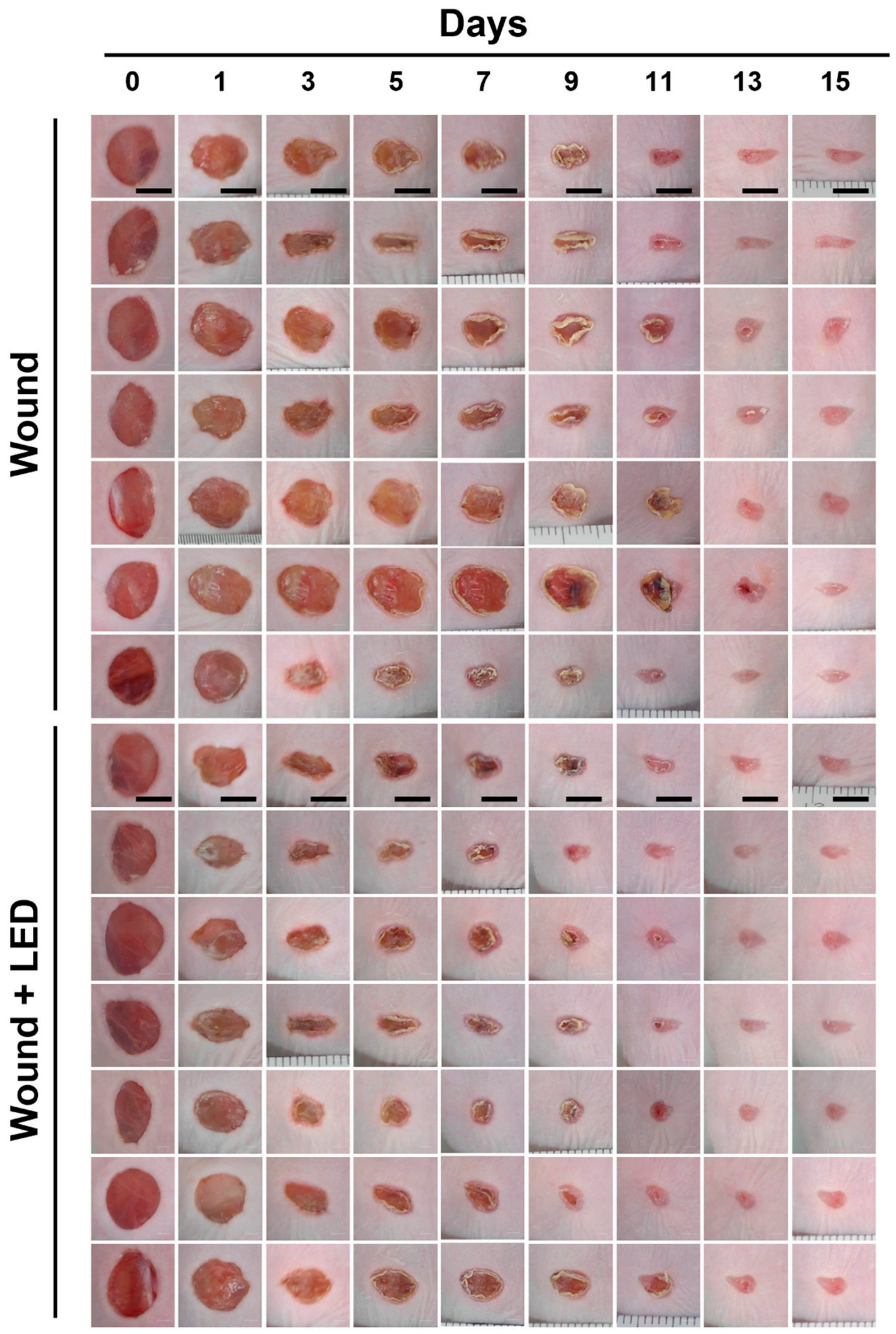

Figure S2. Macroscopic images of wound healing progression at indicated time points. Scale bars = 5 mm.
